# Reflections on Uterine Disease, as Connected with Phthisis Pulmonalis

**Published:** 1872-03

**Authors:** Geo. M. McDowell

**Affiliations:** President of the Georgia Medical Association, Barnesville, Georgia


### REFLECTIONS ON UTERINE DISEASE, AS CONNECTED WITH PHTHISIS PULMONALIS.

BY GEO. M. M'DOWELL, M.D., BARNESVILLE, GA.,
President of the Georgia Medical Association.

While many of the ablest minds in the profession are industriously investigating the pathology, therapeutics and surgery of the uterus, and by their efforts are bringing relief to thousands of afflicted females, who, in former times, even under our immediate ancestors, would have been doomed to an untimely death, or a life of continuous suffering more terrible than death itself, it is somewhat remarkable that so little attention has been attracted to a feature in that class of maladies which materially affects the treatment, and should modify the prognosis. I refer to the frequent and close relationship existing between the tuberculous diathesis and diseases of the uterus, especially chronic diseases of the *cavity* of that organ. Some of the most popular and advanced writers on gynæcology fail to mention it in connection with the etiology of uterine disease; while the slight allusion to it by others would seem to indicate that, although it has not escaped their observation, its importance is not fully appreciated. Dr. Gardner, in his American edition of Scanzoni, gives it a passing recognition, and Prof. Thomas, in his admirable work on "Diseases of Women," distinctly mentions the "hæmic conditions accompanying phthisis" among the causes of endometritis.

Within the range of my own experience and observation, nothing connected with the causation of disease has more strongly impressed me than the fact that so large a proportion of the cases of inflammation and ulceration of the uterus, which I have seen, were connected with the tubercular diathesis—particularly with developed *pulmonary* phthisis.

Allow me to remark, in this connection, that I consider the distinction between constitutional or hereditary and accidental or inflammatory phthisis as *almost* established. Recognizing this distinction, there has *seemed* to be greater immunity from the *severity*, but not from the *frequency*, of uterine disease in the latter (accidental) than in the former (hereditary)—probably in consequence of the septic influences more severely affecting the crasis of the blood (hæmatosis) than in the form of phthisis resulting from inflammatory affections of the lungs, *without* the "hereditary taint."

Many of the old writers have recorded the fact that phthisis affects, in various ways, the menstrual function. Laënnec long ago remarked that the catamenia were "generally defective or absent at an early stage of phthisis." But it appears they did not perceive, or did not deem worthy of record, the fact which I have repeatedly observed: that many of the most extraordinary and intractable irregularities of the catamenia occur in females who, years afterward, become the subjects of phthisis, but who, at the time of the occurrence of these irregularities, were apparently in good health otherwise. Having been continuously engaged in the same community since commencing the practice (seventeen years ago), I have been enabled to watch the progress of many of these cases in families of known tubercular taint. In some of these families, among several sisters, one or more of less vigorous constitutions have been, during their girlhood, the subject of oftrecurring menorrhagia, suspended menstruation, dysmenorrhœa, etc. After marriage, my fears and expectations have seldom failed of realization in having them on the list of my patients with the multiform *uterine diseases*, and *subsequently* with progressive phthisis. Thus have I, again and again, traced, and with suppressed fears tacitly *predicted* the insidious march of this frightful scourge from those slight *but too frequent* manifestations in the catamenial irregularities of the buoyant, hopeful and unsuspecting young lady, through the stages of uterine inflammation and ulceration in the matron, to its culmination in the dreaded "consumption."

I believe but few married women have died of phthisis who had not suffered with severe inflammation or ulceration of some portion of the uterus, and probably not many unmarried ones. Statistical information, published in that valuable old book, "Louis on Phthisis," revealed the fact, that in a given number of males and females suffering with phthisis, ulcerations of the trachea, larynx, epiglottis and bronchia were found much more frequently *in the males than in the females*. But Louis, and all the learned authors who so far as I know have commented on it, found it "impossible to say why." Modern gynæcology is *revealing the secret* in the large number of phthisical females who have ulceration of the uterus, signally elucidating the difficulty that environed our fathers in accounting for the discrepancy! It was long since remarked that, in tuberculous patients, ulcerations were more frequent in the intestines than tubercles. Why not likewise in the uterus? Modern microscopists have verified the observations of the older writers, that tuberculous matter has at times been found in the uterus and ovaries.

It is easy to believe, that on account of the nature of the uterus—the peculiarity of its formations, the fluxional vicissitudes and the diversity of exciting and irritating influences to which it is subject—it would become the favorite seat of those ulcerations which, in consumptive males, are found in the air-passages and alimentary canal.

The pathological facts in the case demand our assent. In consequence of the great impressibility of the nerves of the uterus, a neuropathic condition is frequently induced; and under the excitement, the secretions may be greatly increased and vascular activity developed—resulting in inflammation, ulceration, and the aplastic and cacoplatic deposits that belong to the tubercular and strumous diathesis.

These conditions may exist, too, when the deposit in the lungs is not sufficient to give rise to marked symptoms, and when there is no obvious cachexia. Thus we may fail to appreciate the serious character of the trouble in hand, and be led to make a prognosis that will not be sustained by subsequent developments.

These are the most intractable and unsatisfactory cases encountered in practice. They may be greatly alleviated, and many will be *cured*, but only to return, time after time, as the constitutional malady steadily extends its ravages; while the majority continually disappoint the hopes of the gynæcologist by repeated relapses after periods of improvement.

My observation leads me to the conclusion, that three-fourths of the cases of sterility occurring among respectable married women in this country are the result of cervical or corporeal endometritis in persons who, sooner or later, are subjects of phthisis. Nor can this sterility be regarded as a misfortune, when we remember that the effects of rapid child-bearing, in phthisical females, are almost invariably to hurry them to the grave. Nevertheless, the distressing effects of disease of the womb are so great as to demand the best efforts of the physician for their relief, even at the *hazard of a cure.*

I have fancied that these phthisical patients must have formed a large element in the clinical experience of Scanzoni, when so able and renowned an author could be constrained to say: "We do not remember a single case where we have been able to cure an abundant uterine leucorrhœa of several years' standing." When he shall have had a larger experience in the use of the *hard rubber syringe*, the medicated "cloth tent" of Dr. Taliaferro, and other improved modes of treatment, I doubt not he will be able to publish more satisfactory results—provided his patients be not of the unfortunate class under consideration.

With regard to the treatment of what I shall term "Uterine Phthisis," I would merely suggest the importance of early attention to the constitutional remedies, including all the hygienic and therapeutical agents that are known to stay the progress of phthisis by supporting the *stamina.* In connection with these, we should not fail to resort to local treatment—for, with all the means and appliances which of late years have signalized the progress in gynæcology, we cannot fail to greatly ameliorate the sufferings, though we may often fail of permanent relief.

I will not weary you with tedious details of cases, though I have some statistics concerning the frequent concomitance of uterine disease with phthisis, which I will furnish you at some future time, together with the result of the combined treatment above indicated on these cases. Let it suffice, at present, to give you brief notes of two or three representative cases.

Since recognizing the importance of the distinctions set forth in this paper, it has been my custom to prescribe certain articles of the class of reputed *alteratives*—or what my friend, Prof. J. G. Westmoreland, would more properly term *catalytics*—to dissolve and facilitate the removal of deposits of low organization (cacoplastic), which I feel quite sure they *do accomplish*, to some degree, both in the lungs and *uterus.* My confidence is limited to a small number of these—arsenic, iodine, bromine, and cod-liver oil. I use iodine locally, and iodide of potassium by the stomach.

Miss M———, æt. eighteen; brought to me by her father from a neighboring county. Had symptoms of "womb disease" for six months, with increasing severity. Satisfied that a physical examination was necessary, I instituted it, and found leucorrhœa, with cervical engorgement and slight prolapsus. So soon as I could use a speculum of sufficient size, applied leeches, followed by argent. nit. iodine, carbolic acid, etc., on tents and cotton-wrapped probe, at suitable intervals, with such attention to complications as symptoms suggested. The treatment would give more or less relief, after each of numerous relapses for three months.

Though members of the family had died of consumption, I had not suspected the patient, till finally, on interrogation,

I found there was a slight cough of some weeks' standing. Auscultation and percussion gave satisfactory evidence, to my mind, of the existence of tubercles. Gave her oleum morrhuæ, with arsenic, and continued the same local treatment. Improvement marked and rapid; relapses less frequent, and always followed by prompt improvement under above treatment, but not under local treatment tried *alone*.

Mrs. P———, æt. thirty-five; mother of two children—the youngest twelve years old. Had chronic endometritis, well-marked by usual symptoms; had, also, dismenorrhœa, cough, mumular sputa, pains in chest. Several members of the family died with phthisis. Had been *twice* cured of uterine disease, within the last five years, by different physicians; returned in a few months. Tried intra-uterine medication, in usual manner, with slow progress, for three months. Gave her oleum morrhuæ, with arenic, and continued same local treatment. Improvement immediate and rapid, both in lungs and uterus, for three months, when the metritis seemed cured. Left off all treatment but oleum morrhuæ, which was continued four months longer, and then discontinued. At present time (six months after leaving off), the original trouble has returned in lungs and womb, and treatment renewed.

Mrs. F———, æt. thirty-two; married twelve years; has had no children. Chronic endometritis; cough; dyspnœa; vitiated expectoration; obvious tubercles in both lungs. Treatment of uterine trouble with applications, and injections into *cavity* of iodine, carbolic acid, etc. Some improvement; progress variable and unsatisfactory for many weeks. Prescribed oleum morrhuæ, with potass. iodid.; applied oleum tiglii to chest and hypogaster. Improvement prompt and rapid, and *sustained* for five months. Left off treatment.

In two or three months, pulmonary and uterine symptoms returned with increased impetus. Renewed oleum morrhuæ, with potass. iodid., *without* local treatment to the uterus. Prompt improvement in lungs and *uterus*.

This case is always worse when off the treatment for a few weeks, but improves *in every respect* on renewal of the cod-liver oil.

